# Contribuições da vitamina D no tratamento de sintomas depressivos e fatores de risco cardiovascular: protocolo de estudo para um ensaio clínico randomizado, duplo-cego e controlado por placebo

**DOI:** 10.1186/s13063-019-3699-3

**Published:** 2019-10-11

**Authors:** Catarina Magalhães Porto, Tatiana de Paula Santana da Silva, Everton Botelho Sougey

**Affiliations:** Neuropsiquiatria e Ciências do Comportamento, Universidade Federal de Pernambuco (UFPE), Av. Moraes Rego, 1235, Cidade Universitária, Recife, PE CEP: 50670-901 Brasil

**Keywords:** Vitamina D, Sintomas depressivos, Transtorno depressivo maior, Doença cardiovascular, Ensaio clínico

## Abstract

**fundo:**

A depressão é uma das principais causas de incapacidade crônica em todo o mundo e um importante fator de risco cardiovascular, aumentando o risco relativo de doença arterial coronariana, bem como as taxas de morbimortalidade cardiovascular. Concomitantemente à alta prevalência de depressão, houve uma redução na exposição à luz solar com o aumento da urbanização e do uso de protetores solares, o que levou a uma redução nos níveis séricos de 25-hidroxivitamina D. Portanto, este artigo descreve uma protocolo para um ensaio clínico com o objetivo de avaliar os efeitos da suplementação de vitamina D na depressão e fatores de risco cardiovascular para contribuir com evidências sobre a influência potencial da suplementação na regulação do humor.

**Métodos:**

Este estudo de protocolo foi orientado pelos itens de protocolo padrão: recomendações para ensaios intervencionistas. Um ensaio clínico randomizado, controlado por placebo, duplo-cego será realizado envolvendo 224 adultos (faixa etária de 18 a 60 anos) com depressão que estão tomando antidepressivos e não têm histórico de suplementação de vitamina D, comorbidades psiquiátricas, doença renal crônica, hipercalcemia, ou neoplasia. Os participantes serão recrutados nos ambulatórios psiquiátricos de duas universidades do nordeste do Brasil. Os participantes elegíveis que fornecerem consentimento por escrito serão designados aleatoriamente para o grupo de intervenção (*n* = 112; suplementação de vitamina D 50.000 UI por semana durante 6 meses) ou para o grupo controle (*n* = 112; placebo tomado semanalmente por 6 meses). Medidas para monitorar sintomas depressivos, exames clínicos e exames laboratoriais para avaliar fatores de risco cardiovascular e níveis séricos de vitamina D serão realizadas antes e após o período de intervenção.

**Discussão:**

Até onde sabemos, este será o primeiro ensaio clínico com o objetivo de testar a eficácia da suplementação de vitamina D na redução do risco cardiovascular e como um adjuvante à terapia da depressão por um período prolongado (6 meses). Os resultados contribuirão para a compreensão dos efeitos terapêuticos da suplementação de vitamina D no tratamento da depressão e podem ajudar a orientar políticas públicas direcionadas à suplementação de vitamina para a redução do risco cardiovascular.

**Registro de teste:**

Registro Brasileiro de Ensaios Clínicos, RBR-6yj8sj/ Número Universal de Ensaios (UTN) U1111-1217-9237. Registrado em 23 de julho de 2018.

## fundo

A depressão é uma das principais causas de incapacidade em todo o mundo, com um relatório de mais de 300 milhões de indivíduos afetados em 2015, o que corresponde a 4,4% da população global [1]. A depressão também é considerada um fator de risco cardiovascular, aumentando o risco relativo de doença arterial coronariana, bem como as taxas de morbimortalidade cardiovascular [2–4]. Como a depressão é um distúrbio complexo que provavelmente tem vários subtipos e etiologias, o calcitriol (vitamina D) pode desempenhar um papel importante nessa condição [5].

Os receptores de vitamina D foram identificados em áreas do cérebro envolvidas com a depressão, como o córtex pré-frontal, o hipotálamo e a substância negra. Portanto, esta vitamina é considerada um neurosteróide. Verificou-se também que a vitamina D aumenta a expressão de genes que codificam a tirosina hidroxilase, que é um precursor da dopamina e da norepinefrina [5]. Além disso, o calcitriol pode fornecer proteção significativa contra os efeitos da redução de neurotransmissores (dopamina e serotonina) em doses neurotóxicas de metanfetamina. Verificou-se que o calcitriol desempenha um papel importante nos neurônios em estudos in vitro e in vivo e tem a capacidade de regular fatores tróficos e proteger contra várias lesões [6].

Muitos estudos sugeriram que distúrbios psiquiátricos, como esquizofrenia, alcoolismo e depressão, podem estar associados a baixos níveis séricos de 25-hidroxivitamina D, 25 (OH) D [7–13]. Estudos recentes relatam que a deficiência de vitamina D pode estar associada a um aumento nas taxas de depressão de 8 a 14% [14–19].

Além disso, um estudo duplo-cego, controlado por placebo, mostrou que a suplementação de vitamina D por 8 semanas foi benéfica, mas não significativa (*p* = 0,06) na redução dos sintomas de depressão, demonstrando uma possível tendência dessa ação hormonal no tratamento da depressão. No entanto, os autores relataram uma melhora na insulinemia e homeostase das células β pancreáticas, interferindo também na capacidade antioxidante total do plasma [20]. Meta-análises recentes conduzidas de acordo com os itens de relatório preferenciais para análises sistemáticas e meta-análises (PRISMA) relatam uma redução estatisticamente significativa nos sintomas depressivos e uma redução clinicamente significativa na depressão após a suplementação de vitamina D [21, 22].

Este artigo apresenta o protocolo para um ensaio clínico randomizado, controlado por placebo, duplo-cego, com o objetivo de avaliar os efeitos terapêuticos da vitamina D (50.000 UI por semana durante 6 meses) na depressão e fatores de risco cardiovascular em pacientes com depressão em tratamento nas clínicas psiquiátricas de dois hospitais universitários no nordeste do Brasil em comparação com um grupo controle (placebo uma vez por semana durante 6 meses). A hipótese é que a suplementação de vitamina D é capaz de melhorar os sintomas depressivos e diminuir o risco de suicídio e o risco cardiovascular em pacientes com depressão.

## Métodos

**Figura 1:**
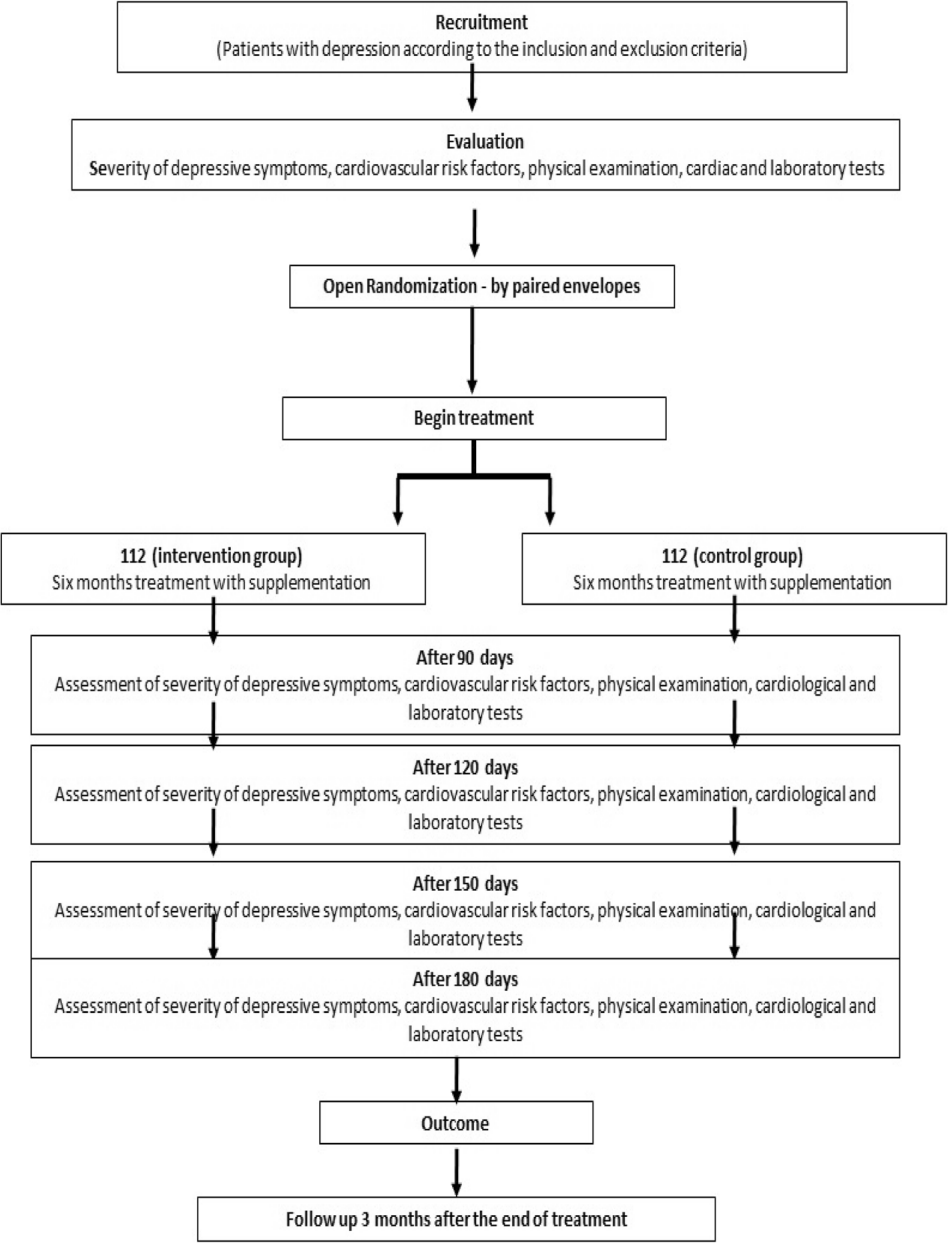
Fluxograma do estudo

Este estudo de protocolo foi orientado pela declaração de itens de protocolo padrão: recomendações para ensaios intervencionistas (SPIRIT) (consulte a Fig. 1a lista de verificação do SPIRIT). O estudo prospectivo, randomizado, controlado por placebo e duplo-cego proposto foi fluxograma do estudo e o arquivo adicional 1, a lista de verificação do SPIRIT). O estudo prospectivo, randomizado, controlado por placebo e duplo-cego proposto foi devidamente registrado (http://www.ensaiosclinicos.gov.br/rg/ com o número de registro do estudo RBR-6yj8sj e o Número Universal de Estudo (UTN) U1111-1217 -9237, 23 de julho de 2018).

O ensaio clínico será conduzido de acordo com os preceitos estipulados na Declaração de Helsinque, e o projeto recebeu aprovação do Comitê de Ética em Pesquisa em Humanos da Universidade Federal de Pernambuco (certificado número 36379347717.0.0000.5208 de 01/01/2018). Todos os participantes serão recrutados nos ambulatórios psiquiátricos da Universidade de Pernambuco e da Universidade Federal de Pernambuco. Avaliaremos 224 adultos do sexo masculino e feminino (18 a 60 anos) com depressão sob cuidados de rotina nas clínicas psiquiátricas por um período de 6 meses.

### Critérios de elegibilidade

Indivíduos entre 18 e 60 anos com diagnóstico de depressão (escore> 6 pontos na Escala de Classificação de Depressão de Montgomery-Asberg [23]) serão recrutados independentemente de sexo ou sexo. Os participantes precisarão ter um diagnóstico de depressão fornecido por um psiquiatra e com base na CID-10 [24], como F33.0 (depressão leve recorrente), F33.1 (transtorno depressivo moderado recorrente), F33.2 (transtorno depressivo recorrente grave sem sintomas psicóticos, incluindo depressão maior recorrente, depressão endógena e psicose maníaco-depressiva). forma depressiva sem sintomas psicóticos), F33.3 (transtorno depressivo grave com sintomas psicóticos, incluindo depressão maior com sintomas psicóticos, depressão psicótica, psicose maníaco-depressiva na forma depressiva com sintomas psicóticos), F33.8 (outros transtornos depressivos recorrentes) e F33.9 (transtorno depressivo grave recorrente sem sintomas psicóticos, incluindo depressão maior recorrente sem especificação ou depressão unipolar).

Os pacientes podem estar tomando um antidepressivo prescrito por seus psiquiatras. Nenhuma restrição será imposta em relação ao tipo de medicamento antidepressivo. A gravidade da doença pode ser leve, moderada ou grave, conforme avaliado pelo psiquiatra.

Indivíduos com comprometimento cognitivo (determinado pelo Mini-Exame de Saúde Mental [25]), aqueles que já suplementam com vitamina D, aqueles com histórico de comorbidades psiquiátricas, doença renal crônica, hipercalcemia ou neoplasias e mulheres grávidas ou amamentando excluídos do estudo.

### Procedimentos operacionais

Dois farmacêuticos farão a randomização e estarão cientes de quais pacientes estão usando vitamina D e quais estão usando o placebo. Os participantes serão divididos em dois grupos (A e B). O pesquisador principal do ensaio clínico entrevistará todos os participantes e preencherá os prontuários clínicos. Uma enfermeira administrará a vitamina D e o placebo, mas não saberá qual substância está sendo administrada aos participantes. Os pacientes e o investigador desconhecem qual cápsula (vitamina D ou placebo) os pacientes estão tomando. Este procedimento é necessário para evitar viés de observação durante o acompanhamento do laboratório clínico e manter total imparcialidade na avaliação dos efeitos.

Todas as instruções serão precisas e escritas na forma de um manual para garantir a execução de todos os procedimentos determinados para o ensaio clínico, incluindo recrutamento de pacientes, alocação para os grupos de estudo, administração da intervenção, sistemas de registro, critérios para interromper a intervenção, etc. Todas as atividades a serem executadas durante o ensaio clínico serão previamente estabelecidas na forma de uma lista de tarefas distribuída à equipe de investigação.

A avaliação inicial consistirá em preenchimento da ficha de identificação, inventário sociodemográfico, ficha médica padronizada com histórico do paciente, exames físicos e cardiológicos, um questionário abordando a prática de atividades que envolvem exposição à luz solar, declaração padronizada de consentimento informado, entrevista estruturada sobre depressão com o uso da Escala de Depressão de Montgomery-Asberg, uma escala que aborda o risco de suicídio e o registro dos resultados de exames laboratoriais e cardiológicos. O pesquisador acompanhará os exames e a administração das escalas de depressão e risco suicida.

Os testes laboratoriais serão realizados no laboratório do hospital em que o estudo será desenvolvido. O sangue venoso (10 ml) será coletado de uma veia periférica para a determinação da glicemia de jejum, colesterol (total e frações), uréia, creatinina, proteínas totais, albumina, homocisteína, proteína C reativa ultrassensível, lipoproteína A, cálcio iônico, e fósforo, bem como níveis hormonais de testosterona total e livre, estradiol, estriol, hormônio luteinizante, hormônio folículo-estimulante, hormônio estimulador da tireóide (TSH), triiodotironina, tetraiodotironina livre, cortisol, concentração de25-hidroxivitamina D e hormônio estimulador da paratireóide na admissão no estudo e a cada 90 dias por um período total de 6 meses. A urina será coletada a cada 90 dias para a determinação de 24 h de calciúria e microalbuminúria. Os seguintes equipamentos de laboratório serão utilizados para as determinações: arquiteto C8000 para concentrações de vitamina D e hormônios e CMD 800 com ISE (Wiener Group) para concentrações bioquímicas.

Exames cardiológicos e de imagem (eletrocardiografia, ecocardiograma Doppler, teste de estresse, monitoração eletrocardiográfica [Holter 24 horas] e ultrassonografia Doppler das artérias carótida e vertebral) serão realizados no hospital em que o estudo será desenvolvido.

Quando os participantes retornarem com os resultados do exame, uma randomização simples (cara ou coroa) será realizada para a alocação aos diferentes grupos: 112 indivíduos no grupo da vitamina D _3_ e 112 no grupo do placebo. Uma enfermeira da clínica administrará a vitamina D _3_ e o placebo. As cápsulas terão o mesmo tamanho e cor (compostas para o ensaio clínico), e o enfermeiro ficará cego para a alocação dos pacientes para os diferentes grupos.

Os pesquisadores avaliarão os participantes a cada 90 dias. Cada avaliação envolverá os exames clínicos e cardiológicos com a eletrocardiografia e os exames laboratoriais selecionados para o estudo. A avaliação final ocorrerá após 6 meses de intervenção. Os participantes serão submetidos a testes psiquiátricos (Escala de Classificação de Depressão de Montgomery-Asberg [23] e Escala de Classificação de Gravidade Suicida de Columbia [26]), exames clínicos e cardiológicos, além de exames laboratoriais, eletrocardiografia, ecocardiograma Doppler, teste de estresse, Ultra-som Holter 24 horas e Doppler das artérias carótidas e vertebrais.

Como a região em que o ensaio clínico será realizado é ensolarada durante todo o ano, com uma ausência de estações climáticas bem definidas, a sazonalidade não exerce influência sobre a exposição à luz solar ou a síntese de vitamina D [27].

### Cálculo do tamanho da amostra

Foram considerados os seguintes para a determinação do tamanho da amostra:

1. O objetivo comparativo será uma melhora no escore de depressão entre os pacientes que tomam e os que não tomam vitamina D. Consideramos as porcentagens de sucesso obtidas em um estudo sobre vitamina D [28], no qual foram encontradas melhorias de 69,2% e 65% na depressão pontuações na escala, respectivamente.
2. Assumimos 5,0% de taxa de erro aceitável, 80,0% de potência e uma taxa de 1,00 entre os dois grupos.

O tamanho da amostra necessário para cada grupo foi de 112 pacientes (total de 224 pacientes). Este cálculo foi realizado usando o programa Epi Info, versão 6.04.

### Redução do risco de viés

A dosagem de vitamina D para suplementação considerada para o estudo (50.000 UI por semana) atende aos critérios pré-estabelecidos em estudos anteriores que apresentaram resultados positivos na redução dos sintomas depressivos [20]. O objetivo de considerar essa variável é minimizar o potencial viés. A vitamina D _3_ é bastante segura quando administrada com a posologia prescrita. Dosagens de até 10.000 UI por dia durante 5 meses não induziram sinais de toxicidade, como hipercalcemia e hipercalciúria [29, 30].

### Coleta e gerenciamento de dados, incluindo metadados

As características e procedimentos adotados para cada grupo de teste são os seguintes:

- Grupo 1 - Intervenção: 112 participantes receberão suplementação de vitamina D _3_ (Addera D3, Laboratório Mantecorp) na dose de 50.000 UI por semana, durante 6 meses.
- Grupo 2 - Placebo: 112 participantes receberão uma cápsula de placebo (composta pelo laboratório farmacêutico da Universidade Federal de Pernambuco) uma vez por semana, durante 6 meses.

Os grupos serão submetidos a cinco avaliações no primeiro dia e após 6 meses de intervenção:

1. *Questionário abordando características sociodemográficas*. O questionário será aplicado aos participantes em formulário de entrevista e preenchido pelo pesquisador. Os seguintes dados serão registrados: número de identificação atribuído ao participante, idade, sexo, escolaridade, estado civil, etnia e religião.
2. *Critérios de Classificação Econômica Brasileira (BECC) - 2011 estabelecidos pela Associação Brasileira de Empresas em Pesquisa* [31]. O BECC é um instrumento de segmentação econômica que utiliza um levantamento das características residenciais (presença e quantidade de itens domésticos de conforto e grau de escolaridade do chefe de família) para diferenciar a população. Pontos são atribuídos a cada característica da família, e o total é usado para classificar os estratos econômicos da classe mais alta para a mais baixa: A1, A2, B1, B2, C1, C2, D, E [31].
3. *Gravidade da depressão*. Isso é determinado através de uma entrevista clínica estruturada. A Escala de Classificação de Depressão de Montgomery-Asberg [23] será administrada no início do estudo e após 6 meses de intervenção.
4. *Avaliação de risco de suicídio*. A Columbia-Suicide Severity Rating Scale [26] será administrada para avaliar a presença e a intensidade de ideação suicida e comportamento suicida.
5. *Fatores de risco cardiovascular*. Estes são (1) hipertensão arterial sistêmica com base nos níveis de pressão arterial sistólica e diastólica [32]; (2) dislipidemia (colesterol total, lipoproteínas de alta e baixa densidade [HDL, LDL] triglicerídeos) [33]; (3) doença arterial coronariana: cálcio coronariano> percentil 100 ou> 75 para idade ou sexo, teste de estresse cardíaco ou cintilografia do miocárdio com sinais de isquemia miocárdica [34]; (4) acidente vascular cerebral (AVC): condição clínica ou exame de imagem sugestivo; (5) doença arterial periférica obstrutiva: índice de pressão tornozelo-braquial <0,9; estenose arterial detectada por ultrassonografia de membros inferiores [35]; (6)aterosclerose carotídea: estenose / espessamento da artéria carótida [33]; (7) insuficiência cardíaca: hipertrofia ventricular esquerda ou redução da função sistólica do ventrículo esquerdo ou disfunção diastólica detectada pelo ecocardiograma Doppler [36]; (8) arritmia: detectada por eletrocardiografia em repouso, Holter de 24 horas ou teste de estresse; (9) diabetes mellitus: redução da tolerância à glicose e resistência à insulina (glicemia de jejum, hemoglobina A1C, teste oral de tolerância à glicose, insulina basal, curva de insulina, avaliação do modelo homeostático) [37, 38]; (10) marcadores séricos de risco cardiovascular (proteína C reativa ultrassensível, homocisteína, lipoproteína A) [33; (11) hipertireoidismo ou hipotireoidismo (TSH ultrassensível, T4 livre, T3 livre) [39, 40]; (12) doença renal crônica: albuminúria, medida pela taxa de filtração glomerular e calculada com base na Colaboração em Epidemiologia da Doença Renal Crônica [41]; (13) hiperparatireoidismo (HPT, fósforo) [42].

### Análise de dados

Os dados serão analisados ​​por meio de medidas estatísticas: valores de média e desvio padrão para variáveis ​​numéricas e frequências percentuais para variáveis ​​categóricas. As comparações intergrupos serão realizadas usando o teste *t* de Student ou Mann-Whitney para variáveis ​​numéricas e o teste qui-quadrado de Pearson para variáveis ​​categóricas. As comparações intragrupo dos valores inicial e final serão realizadas usando o teste *t* de Student pareado ou o teste de Wilcoxon para dados pareados para variáveis ​​numéricas e o teste de McNemar para variáveis ​​categóricas.

Os testes *t* não pareados e emparelhados serão utilizados em situações em que os dados exibem distribuição normal, enquanto os testes Mann-Whitney e Wilcoxon para variáveis ​​emparelhadas serão utilizados em situações em que a normalidade foi rejeitada. O teste de Shapiro-Wilk será usado para determinar a normalidade dos dados, e o teste *F* de Levene será usado para determinar a igualdade de variâncias.

A margem de erro utilizada nas decisões dos testes estatísticos será calculada como intervalos de confiança de 5% e 95%. Os dados serão inseridos em uma planilha do Excel e o Statistical Package for the Social Sciences (SPSS versão 23) será usado para todos os cálculos estatísticos.

Um estatístico realizará os cálculos e análises estatísticas dos participantes que tomam as substâncias A e B. O cegueira só será eliminado ao final da análise estatística.

### Considerações éticas

Este ensaio clínico está em conformidade com a Resolução n ° 466/2012 do Conselho Nacional de Saúde do Brasil, que estabelece normas para pesquisas envolvendo seres humanos, e recebeu aprovação do comitê de ética da Universidade Federal de Pernambuco (UFPE) por meio da Plataforma Brasil (certificado). número 79347717.0.0000.5208) em 11 de janeiro de 2018. O recrutamento (triagem de pacientes) foi iniciado em 30 de abril de 2019. Assim, o estudo está atualmente em sua fase inicial (em andamento). O teste clínico (intervenção) deve começar em 1º de outubro de 2019, conforme estipulado no registro: RBR-6yj8sj / UTN U1111-1217-9237.

### Resultados primários

Os principais resultados do estudo são:

1. Efeitos terapêuticos da vitamina D na depressão, avaliados pela Escala de Depressão de Montgomery-Asberg
2. Efeitos terapêuticos da vitamina D nos fatores de risco cardiovascular, avaliados usando o sistema de pontuação proposto pelo American College of Cardiology (ACC) e pela American Heart Association (AHA) [43, 44] para a prevenção primária do risco de infarto agudo do miocárdio, morte por doença cardíaca coronária, insuficiência cardíaca e derrame fatal e não fatal em um período de 10 anos. O novo sistema de pontuação fornece estimativas específicas com base na idade atual, sexo, raça, pressão arterial, colesterol total, colesterol HDL, colesterol LDL, diabetes, tabagismo (fumante atual, ex-fumante ou nunca fumado), no tratamento de doenças arteriais sistêmicas. hipertensão, no tratamento com estatinas e no tratamento com aspirina. Após incluir esses dados, a calculadora estima o risco de doenças cardiovasculares.doença nos próximos 10 anos, classificada como baixa (<5%), limítrofe (5 a 7,4%), intermediária (7,5 a 19,9%) ou alta (≥ 20%).

### Resultados secundários

Os resultados secundários são:

1. Efeitos terapêuticos da vitamina D na redução do risco de suicídio, avaliados usando a Escala de Classificação de Gravidade do Suicídio da Columbia
2. Redução dos fatores de risco cardiovascular, conforme descrito na subseção “Coleta e gerenciamento de dados, incluindo metadados”.

### Plano de análise e mensuração dos resultados

Os resultados a serem medidos e analisados ​​são os seguintes:

1. Hipertensão: redução da pressão arterial sistólica ou diastólica em 10 mmHg [45]
2. Dislipidemia: redução do colesterol total em 100 mg / dL; aumento de HDL em 10 mg / dL; redução de triglicerídeos em 50 a 100 mg / dL [33]
3. Doença arterial coronariana: melhora da isquemia miocárdica; redução no escore de cálcio coronário [34]
4. Acidente vascular cerebral
5. Doença arterial periférica obstrutiva: melhora no índice de pressão tornozelo-braquial <0,9; estenose arterial detectada pelo ultra-som de trombose venosa profunda dos membros inferiores [35]
6. Aterosclerose carotídea: redução na espessura íntima-média ou no grau de estenose carotídea [33]
7. Insuficiência cardíaca: redução no grau de hipertrofia ventricular esquerda ou aumento da função sistólica do ventrículo esquerdo ou melhora e / ou reversão da disfunção diastólica, detectada pelo ecocardiograma Doppler [36]
8. Arritmia: redução no número de eventos de arritmia, detectados pelo teste de suporte ou estresse de 24 horas
9. Diabetes mellitus: redução da tolerância à glicose e resistência à insulina; melhorias na glicemia de jejum, hemoglobina A1C, teste de tolerância à glicose oral, insulina basal, curva de insulina, avaliação do modelo homeostático [37, 38]
10. Marcadores séricos de risco cardiovascular: reduções na proteína C-reativa ultrassensível, homocisteína e lipoproteína A [33];
11. Hipertireoidismo ou hipotireoidismo: melhora nos níveis séricos de TSH ultrassensível, T4 livre e T3 livre [39, 40]
12. Doença renal crônica: redução da albuminúria, medida pela taxa de filtração glomerular e calculada com base na colaboração na Epidemiologia da Doença Renal Crônica [41]
13. Hiperparatireoidismo (HPT): redução ou normalização dos níveis séricos de HPT [42].

## Discussão

O objetivo deste protocolo é descrever procedimentos relacionados ao desenvolvimento de um ensaio clínico sobre os efeitos terapêuticos da vitamina D (50.000 UI por semana durante 6 meses) na depressão e nos fatores de risco cardiovascular em pacientes com depressão em atendimento em clínicas psiquiátricas de dois universidades em comparação com um grupo controle que recebeu um placebo uma vez por semana durante o mesmo período de tempo.

Foi demonstrado que a suplementação de vitamina D em pacientes com depressão melhora os sintomas depressivos, como demonstrado em três revisões sistemáticas [21, 22, 45]. A suplementação de vitamina D teve um efeito moderado estatisticamente significativo em pacientes com sintomas depressivos clinicamente significativos ou transtorno depressivo [22]. Em outra revisão sistemática, a metanálise de todos os estudos sem falhas demonstrou uma melhora estatisticamente significativa na depressão após a suplementação de vitamina D, com um tamanho de efeito comparável ao do medicamento antidepressivo [18]. Com o objetivo de avaliar o potencial dos nutracêuticos, incluindo a vitamina D, como adjuvantes à terapia antidepressiva, uma importante revisão sistemática e metanálise realizada em 2016 obteve resultados positivos, sugerindo a ação moduladora dos nutracêuticos em relação a várias vias neuroquímicas envolvidas na depressão [46].

A combinação de vitamina D e tratamento antidepressivo tradicional pode levar à otimização da terapia para depressão com maior tolerabilidade e sem efeitos colaterais adicionais. Além disso, os efeitos terapêuticos da vitamina D nos fatores de risco cardiovascular podem ter impacto no risco cardiovascular.

A toxicidade da vitamina D é uma condição rara causada por uma overdose de vitamina D, na qual concentrações marcadamente altas de 25 (OH) D (> 150 ng / ml) são acompanhadas por hipercalcemia grave, hipercalciúria e hipoparatireoidismo, com muito baixo ou até indetectável paratormônio (PTH) [4]. Essa condição é normalmente observada em pacientes que recebem doses maciças de vitamina D (50.000 a 1.000.000 UI) diariamente por vários meses a anos [47, 48].

A hipervitaminose D e a hipercalcemia podem ter uma etiologia exógena, provocada pelo uso excessivo ou descontrolado de altas doses orais de vitamina D ou de seus análogos ou metabólitos. Eles também podem ser endógenos devido à produção excessiva de 1,25 (OH) 2D em doenças granulomatosas, como sarcoidose, tuberculose, linfoma e hipercalcemia infantil idiopática [49]. Mutações no gene 1,25 (OH) 2D (OH) 2 do citocromo P450 D-24-hidroxilase (*CYP24A1*) constituem outra causa de toxicidade da vitamina D e hipercalcemia [50].

De acordo com o Institute of Medicine (IOM) e a Endocrine Society, a toxicidade aguda da vitamina D é extremamente rara na literatura, e outros fatores, como a ingestão de cálcio, podem afetar o risco de desenvolver hipercalcemia e toxicidade da vitamina D. Independentemente de fatores de risco adicionais para toxicidade da vitamina D, muitos estudos oferecem evidências de que a vitamina D é provavelmente uma das vitaminas lipossolúveis menos tóxicas e é muito menos tóxica que a vitamina A [51, 52].

Considerando os comentários anteriores, a dosagem de vitamina D proposta para o estudo (50.000 UI por semana) atende aos critérios estabelecidos em um estudo anterior, que relatou resultados positivos em termos de redução dos sintomas depressivos [20]. O objetivo do uso dessa dosagem é minimizar possíveis vieses. O risco de toxicidade é mínimo, pois doses de até 10.000 UI por dia (70.000 UI por semana) durante 5 meses não induziram sinais de toxicidade, como hipercalcemia e hipercalciúria, em um estudo anterior [29].

Os resultados do ensaio clínico proposto podem contribuir para a compreensão dos efeitos terapêuticos da suplementação de vitamina D na depressão e no risco de suicídio. Este estudo também pode demonstrar a importância de investigar os níveis séricos de vitamina D em pacientes com depressão. Com relação ao risco cardiovascular, os resultados do estudo poderiam auxiliar na implementação da avaliação dos fatores de risco cardiovascular em pacientes com depressão, na tentativa de reduzir as taxas de morbimortalidade nessa população. Outro benefício potencial deste ensaio clínico é a contribuição para políticas públicas que abordam a importância dos níveis séricos de vitamina D para a identificação de pacientes com deficiências nessa vitamina e promovem sua suplementação para a prevenção de eventos cardiovasculares, depressão e suicídio.

Os resultados e conclusões deste estudo clínico serão enviados para simpósios nacionais e internacionais, bem como para revistas revisadas por pares.

### Ética e propriedade intelectual

Este ensaio clínico recebeu aprovação do Comitê de Ética em Pesquisa em Humanos da Universidade Federal de Pernambuco (certificado número 2.464.997 em 1 de novembro de 2018).

### Acesso, reutilização e compartilhamento de dados

Os conjuntos de dados utilizados e analisados ​​durante o estudo serão disponibilizados pelo autor através de qualquer solicitação razoável.

### Armazenamento, curadoria e preservação a longo prazo

Os conjuntos de dados utilizados e analisados ​​durante o estudo estarão sob a custódia do pesquisador responsável pelo projeto.

### Limitações

O estudo será realizado em apenas duas universidades públicas. Os participantes também precisarão visitar os ambulatórios semanalmente para receber o medicamento, o que pode levar ao absenteísmo. Para contornar esse problema, um membro da equipe de pesquisa pode visitar a casa do paciente nos casos em que não aparece nas clínicas.

Os medicamentos antidepressivos serão determinados pelo psiquiatra que presta cuidados ao paciente. Assim, os pesquisadores deste ensaio clínico não terão controle sobre o tipo ou dosagem dos antidepressivos prescritos. Essa falta de padronização dos medicamentos antidepressivos pode ser uma fonte de viés na pesquisa.

### Status da avaliação

Para comprovação, consulte http://www.ensaiosclinicos.gov.br/rg/. O julgamento está registrado como número RBR-6yj8sj / UTN U1111-1217-9237 (23 de julho de 2018). O recrutamento (triagem de pacientes) foi iniciado em 30 de abril de 2019. Assim, o estudo está atualmente em sua fase inicial (em andamento). O teste clínico (intervenção) deve começar em 1º de outubro de 2019, conforme estipulado no registro (RBR-6yj8sj / UTN U1111-1217-9237).

## Data Availability

Não aplicável.

## Informação suplementar

**Arquivo adicional 1. Lista de** verificação do SPIRIT 2013: itens recomendados para tratar em um protocolo de ensaio clínico e documentos relacionados. (DOC 123 kb)

## Abreviações

HPT: Hiperparatireoidismo
PRISMA: Itens de relatório preferidos para revisões sistemáticas e metanálises
ESPÍRITO: Itens de protocolo padrão: recomendações para ensaios intervencionistas
SPSS: Pacote Estatístico para as Ciências Sociais
UFPE: Universidade Federal de Pernambuco
UPE: Universidade de Pernambuco

## Informação suplementar

**Informações adicionais** acompanham este artigo em 10.1186/s13063-019-3699-3.

## Aprovação ética e consentimento em participar

O estudo foi realizado de acordo com a Declaração de Helsinque e recebeu aprovação do Comitê de Ética em Pesquisa em Humanos da Universidade Federal de Pernambuco (certificado 2.464.997 em 01/01/2018). O protocolo do estudo também foi registrado retrospectivamente no registro brasileiro de ensaios clínicos (http://www.ensaiosclinicos.gov.br/rg/) com o número RBR-6yj8sj / UTN U1111-1217-9237 (23 de julho de 2018). O consentimento informado será obtido de todos os participantes do estudo.

## Consentimento para publicação

Todos os participantes concordaram com a publicação dos resultados.

## Interesses competitivos

Os autores declaram que não têm interesses concorrentes.
