# Correction to: Contributions of Vitamin D in the management of depressive symptoms and cardiovascular risk factors: study protocol for a randomized, double-blind, placebo-controlled clinical trial

**DOI:** 10.1186/s13063-019-3862-x

**Published:** 2019-11-13

**Authors:** Catarina Magalhães Porto, Tatiana de Paula Santana da Silva, Everton Botelho Sougey

**Affiliations:** Federal University of Pernambuco, UFPE, Recife, Pernambuco Brazil

**Correction to: Trials (2019) 20:583**

**https://doi.org/10.1186/s13063-019-3699-3**

An error occurred during the publication of the original article [1] which led to the text being incorrectly converted into Portuguese.

The Publisher apologizes for this error and any confusion caused.

The original article has also been updated.
